# Neuroprotective Evaluation of *Murraya* Carbazoles: In Vitro and Docking Insights into Their Anti-AChE and Anti-Aβ Activities

**DOI:** 10.3390/molecules30153138

**Published:** 2025-07-26

**Authors:** Himadri Sharma, Niti Sharma, Seong Soo A. An

**Affiliations:** Department of Bionano Technology, Gachon Bionano Research Institute, Gachon University, 1342 Seongnam-daero, Sujeong-gu, Seongnam-si 461-701, Gyeonggi-do, Republic of Korea; himadri@gachon.ac.kr

**Keywords:** carbazole, neuroprotection, acetylcholinesterase, anti-Aβ fibrilization, molecular docking, ADME/T properties

## Abstract

The present study investigated the neuroprotective potential of the *Murraya* carbazole derivatives murrayanol, mahanimbine, murrayafoline A, and 9-methyl-9H-carbazole-2-carbaldehyde using in silico and in vitro assays. The pharmacokinetic properties and potential toxicity (ADME/T) of the carbazole derivatives were assessed to evaluate their prospects as up-and-coming drug candidates. Molecular docking was used to investigate the interactions of the compounds with Aβ (PDB: 1IYT, 2BEG, and 8EZE) and AChE receptors (PDB: 4EY7 and 1C2B). The results from the in vitro assays were used to validate and support the findings from the in silico assays. The compounds demonstrated significant inhibition of acetylcholinesterase (AChE), a key target in neurodegenerative disorders. Murrayanol and mahanimbine presented superior inhibitory activity (IC_50_ ~0.2 μg/mL), outperforming the reference drug, galantamine. The inhibition mechanisms were competitive (murrayanol, murrayafoline A, and 9-methyl-9H-carbazole-2-carbaldehyde) and non-competitive (mahanimbine), supported by low Ki values and strong docking affinities. The compounds also proved effective in reducing Aβ fibrillization (murrayanol: 40.83 ± 0.30%; murrayafoline A: 33.60 ± 0.55%, mahanimbine: 27.68 ± 2.71%). These findings highlight *Murraya* carbazoles as promising scaffolds for multifunctional agents in AD therapy. Further optimization and mechanistic studies are warranted to advance their development into clinically relevant neuroprotective agents.

## 1. Introduction

*Murraya koenigii*, a medicinal plant within the Rutaceae family, has been widely used in Indian and Chinese traditional medicinal practices for centuries [1]. Extensive phytochemical investigations identified a diverse array of potential biologically active compounds within the plant, including terpenoids, alkaloids, flavonoids, coumarins, polyphenols, and essential oils [2]. Among these, alkaloids, particularly, carbazole derivatives, are considered significant contributors to the plant’s diverse medicinal attributes. Numerous studies reported the cytotoxic activity of several *M. koenigii* compounds, pointing towards their potential utility in anti-cancer applications [2].

Key carbazole derivatives from *M. koenigii* are mahanimbine, murrayafoline, 9-formyl-3-methyl carbazole, and murrayanol. Mahanimbine revealed its antioxidant, cytotoxic, and anti-diabetic activities. Murrayafoline is known for its anti-inflammatory and cytotoxic activities, and 9-formyl-3-methyl carbazole exhibits an antioxidant activity. Lastly, murrayanol also exerts an anti-inflammatory activity [2,3,4]. 

Interestingly, a recent report highlighted *M. koenigii*’s impact on cognitive function, demonstrating improved memory and learning abilities in rats with alloxan-induced cognitive dysfunction after a treatment with ethanolic and aqueous extracts of the plant [5]. Cumulative findings suggest that the carbazole derivatives from *M. koenigii* are substantially promising for pharmaceutical drug development, particularly in the fields of cancer and cognitive therapy.

To comprehensively elucidate the role and mechanisms of action of these carbazole derivatives in neuroprotection, four key compounds, i.e., murrayanol, mahanimbine, murrayafoline A, and 9-methyl-9H-carbazole-2-carbaldehyde, were selected for the current study.

Our investigation encompassed a rigorous series of in vitro and in silico assays designed to assess their neuroprotective potential. Specifically, their inhibitory effects on acetylcholinesterase (AChE) and amyloid-beta (Aβ) fibrillization were evaluated in vitro. A molecular docking study was subsequently performed to gain insights into protein–ligand interactions. In silico absorption, distribution, metabolism, excretion, and toxicity (ADMET) analyses were followed by an in silico blind docking approach to determine their ligand binding affinities for AChE and Aβ-species.

## 2. Results

### 2.1. Structural Physiognomies

**Figure 1:**
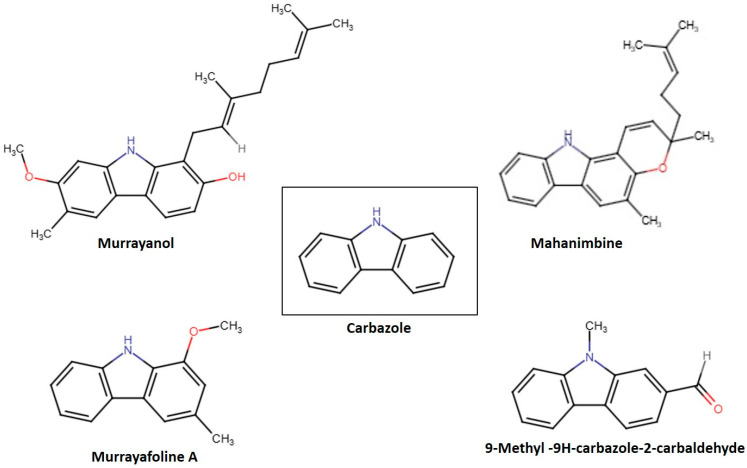
Structures of some *Murraya* carbazole derivatives.

Variations in functional groups, such as hydroxyl, aldehyde, and methyl groups, defined the reactivity of each compound. These structural variations led to unique biological activities, hydrophilicity, and pharmacokinetic characteristics. Figure 1 displays the chemical structures of the carbazole derivatives in this study. Murrayanol (molecular formula, C_24_H_29_NO_2_; molecular weight, 363.5) possesses a complex chemical structure consisting of a carbazole skeleton characterized by hydroxyl (-OH) groups, which enhances its polarity and possible biological activity, and a prenyl side chain. The existence of several chiral centers within its molecule results in different stereoisomers, greatly influencing its pharmacological interactions. On the other hand, mahanimbine (molecular formula, C_23_H_25_NO; molecular weight, 331.5) has a dibenzo cyclooctadiene framework. It has a pyran ring (-O-CH_2_-O) at the 3,4 position and a methylene group (-CH_2_) at the 7 position. The distinctive bicyclic framework results in an increased stiffness in the molecular shape, setting it apart from the relaxed chiral forms, such as murrayanol. The geometric limitations created by its rings also affect its reactivity with biological targets. Murrayafoline A (molecular formula, C_14_H_13_NO; molecular weight, 211.2) has two substituents attached to the carbazole skeleton: a methoxy (-OCH_3_) at position 1 and a methyl group (-CH_3_) at position 3. The methoxy group may affect lipophilicity and electron density, affecting the molecule’s pharmacokinetics. The methyl (-CH_3_) group slightly alters the steric hindrance and hydrophobic interactions that influence its chemical properties and possible pharmacological uses. 9-Methyl-9H-carbazole-2-carbaldehyde (molecular formula, C_14_H_11_NO; molecular weight, 209.2) features a fused aromatic structure that comprises both an N-methyl (-NCH_3_) group and an aldehyde (-CHO) functional group. These properties provide unique electronic characteristics, affecting the molecule’s interactions in biological settings. The aldehyde group at the 2-position adds a level of reactivity that was not observed for the other compounds in this study. 

### 2.2. Drug-Likeness, Toxicity Predictions, and ADME Properties of the Compounds

The preclinical evaluation of the examined carbazole derivatives was performed based on ADME/T (adsorption, distribution, metabolism, excretion, and toxicity) features. The physicochemical parameters were determined based on lipophilicity of the compound (LIPO): CLogP < +5.0; size: 150 g/mol < molecular weight (MW) < 500 g/mol; polarity (POLAR): 20 Å^2^ < topological polar surface area (TPSA) < 130Å^2^; insolubility (INSOLU): −6 < LogS (ESol) < 0; unsaturation (UNSATU): 0.25 < fraction of sp 3 carbon atoms (Fsp3) < 1; and flexibility (FLEX): 0 < number of rotatable bonds (RB) < 9 [6]. A ligand was considered active when two or more of the above conditions were satisfied. Lipinski’s rule of 5 comprises the following criteria: (i) MW < 500 g/mol, (ii) LogP value < 5, (iii) polar surface area (PSA) < 140Å, (iv) RB < 10, and hydrogen bond acceptor/donor < 10/5. Compounds that showed more than one violation of the above criteria were usually not considered orally active drugs. These parameters were assessed using the SwissADME web tool. Both murrayanol and mahanimbine had lipophilicity and solubility values exceeding the specified range of acceptance, and each displayed one violation of Lipinski’s rule. Murrayafoline A and 9-methyl-9H-carbazole-2-carbaldehyde had INSATU values below the specified range. The findings are presented in Table 1. Figure S1 represents the bioavailability radars, consisting of six parameters: lipophilicity, size, polarity, saturation, solubility, and flexibility. The pink zone displays the physicochemical range, making it easier for a quick evaluation of the compound for its drug-likeness.

The ADME properties of the carbazole derivatives are presented in Table 2. The SwissADME tool predicted high gastrointestinal (GI) absorption for all the compounds, but blood–brain barrier (BBB) permeation for murrayafoline A and 9-methyl-9H-carbazole-2-carbaldehyde only. Among the compounds, murrayanol and 9-methyl-9H-carbazole-2-carbaldehyde appeared not to be P-glycoprotein (P-gp) substrates. The results for cytochrome P450 (CYP) interaction showed that all compounds are inhibitors of CYP2C19. Additionally, murrayanol appeared as an inhibitor of CYP2D6, and 9-methyl-9H-carbazole-2-carbaldehyde inhibited CYP1A2. Mahanimbine showed inhibition towards all CYPs, and murrayafoline A showed inhibition towards CYP1A2, CYP2C19, CYP2D6, and CYP3A4. CYP3A4 was inhibited by mahanimbine and murrayafoline A. Cytochrome P450 enzymes are responsible for the metabolism of drugs in our body. If a compound shows inhibition towards all CYP enzymes, this could lead to increased toxicity as a result of drug accumulation, which can increase drug–drug interactions. Figure S2 presents the boiled-egg plot that comprises three parts: yellow yolk (BBB permeability), white region (HIA: human intestinal absorption), and grey region (no HIA/BBB access). This plot graphically represents the drug-likeness of a molecule based on its lipophilicity (fat solubility) and hydrophilicity (water solubility). As observed in Figure S2, murrayafoline A and 9-methyl-9H-carbazole-2-carbaldehyde are present in the yellow region, implying that they are better candidates for oral bioavailability than murrayanol and mahanimbine. Murrayanol and mahanimbine are present in the white region, implying that they may not be good neuroprotective drug candidates due to their poor solubility/absorption.

The ProTox3.0 web tool was used for the toxicity prediction study. It employs machine learning and similarity-driven methods to categorize compounds using established toxicological information. Using web tools for toxicity analysis is termed green toxicology, since animal testing and the total amount of chemicals used during toxicity testing are reduced [7]. Table 3 presents the results of the toxicity of the carbazole derivatives based on hepatotoxicity, neurotoxicity, cytotoxicity, and the toxicity class of the compounds. 

The terms “Active” and “Inactive” refer to the “toxic” and “non-toxic” behavior of the compound for the assessed endpoints. No hepatotoxicity and cytotoxicity were observed for all four carbazole derivatives. Murrayanol and mahanimbine did not show neurotoxicity, whereas the results indicated that murrayafoline A and 9-methyl-9H-carbazole-2-carbaldehyde may be neurotoxic. ProTox3.0 also analyzed the LD_50_ values and toxicity class of the compounds. Based on the lethal dose value, the toxicity class was defined, which indicated the safety of the compounds for oral administration. Murrayanol and mahanimbine belong to class V, indicating that they would be safe for oral administration. 9-Methyl-9H-carbazole-2-carbaldehyde belongs to class IV, indicating its potential harmfulness upon oral administration. Likewise, murrayafoline A belongs to class III, i.e., this compound is toxic if swallowed.

### 2.3. Molecular Docking

For this study, blind docking was performed, as the target pocket dimensions were not known. It helped identify binding sites on the examined receptor proteins, as each entire receptor protein was enclosed within the grid box, allowing the software algorithm to determine the best conformation of the ligand for binding across it. In this study, ligand interactions with two different receptors were studied, i.e., the Aβ receptor (PDB ID: 1IYT; Aβ_1-42_ monomer, PDB ID: 2BEG; Aβ_1-42_ pentamer, PDB ID: 8EZE; Aβ_1-42_ octamer) and the AChE receptor (PDB ID: 4EY7; human recombinant AChE, PDB ID: 1C2B; electric eel AChE).

#### 2.3.1. Docking with the AChE Receptor

The crystal structures of human AChE (4EY7) and electric eel AChE (1C2B) were selected and downloaded from the RCSB PDB website. The binding energies for the ligands are displayed in Table 4. 

Murrayanol displayed a binding affinity of −11 kcal/mol for huAChE (4EY7). Mahanimbine showed the least binding affinity for huAChE (4EY7), at −12.4 kcal/mol, in comparison to other carbazole derivatives. Murrayafoline A showed binding affinities of −9.1 kcal/mol for 4EY7. 9-Methyl-9H-carbazole-2-carbaldehyde displayed a similar binding affinity of −8.8 kcal/mol for 4EY7. 

**Figure 2:**
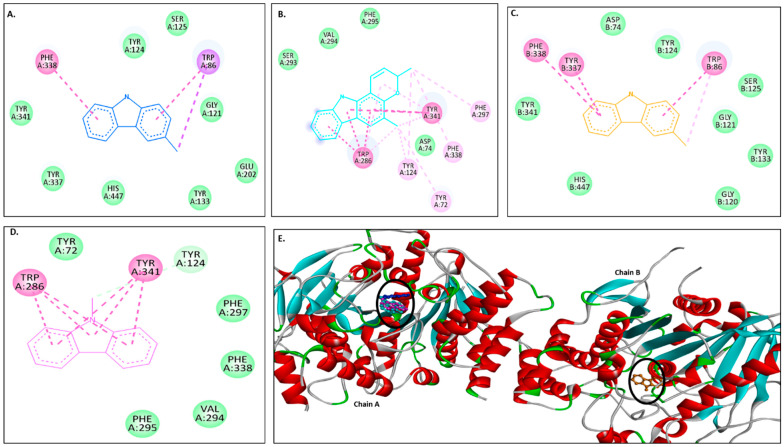
Visual representation of the interactions between ligands and receptor molecule (4EY7). Two-dimensional visualization of the binding interactions of (**A**) murrayanol (dark blue), (**B**) mahanimbine (sky blue), (**C**) murrayafoline A (orange), and (**D**) 9-methyl-9H-carbazole-2-carbaldehyde (pink). (**E**) The marked black zone indicates the binding sites of the ligands in the 3D structure of the protein.

Figure 2A–D show 2D images of carbazole derivatives’ binding interactions with huAChE (4EY7). Figure 2E shows the binding sites in the 3D structure of the protein. Figure S3 displays the 3D binding interactions of the ligand molecules with the receptor. The protein 4EY7 consists of two chains (A and B). As depicted in Figure 2E, murrayanol, mahanimbine, and 9-methyl-9H-carbazole-2-carbaldehyde bind to the protein in chain A, whereas murrayafoline A binds to chain B. Mahanimbine and 9-methyl-9H-carbazole-2-carbaldehyde attach to the same site. Both ligands form a pi–pi (π-π stacked) interaction at TRP286 and TYR341. 9-Methyl-9H-carbazole-2-carbaldehyde forms an H-bond at TYR124. Murrayanol forms a π-π (stacked) interaction at PHE338 and TRP86 and a pi–sigma (π-σ) bond at TRP86. Murrayafoline A interacts with the protein in chain B (Figure 2C), forming π-π (stacked) bonds at PHE338, TYR337, and TRP86, and a π–alkyl bond at TRP86. Van der Waals interactions are also present: murrayanol at SER125, TYR124/133/341/337, HIS447, GLU202, and GLY121; mahanimbine at VAL294, PHE295, SER293, and ASP74; murrayafoline A at ASP74, TYR124/133/341, SER125, GLY121/120, and HIS447; 9-methyl-9H-carbazole-2-carbaldehyde at TYR72/124, PHE297/338/295, and VAL294.

**Figure 3:**
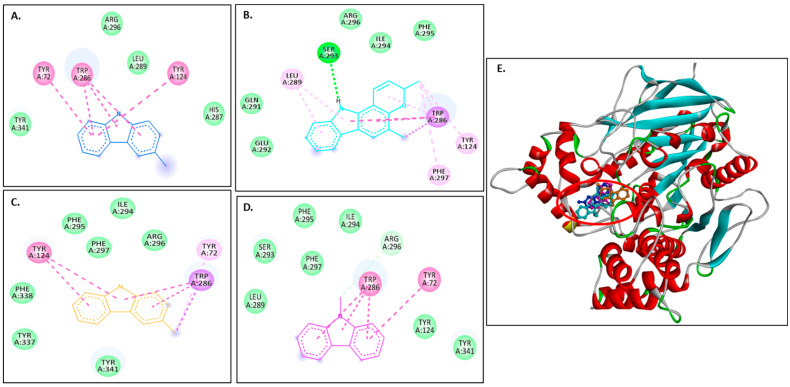
Visual representation of the interaction between ligands and receptor molecule (1C2B). Two-dimensional visualization of the binding interactions of (**A**) murrayanol (dark blue), (**B**) mahanimbine (sky blue), (**C**) murrayafoline A (orange), and (**D**) 9-methyl-9H-carbazole-2-carbaldehyde (pink). (**E**) The marked black zone indicates the binding sites of the ligands in the 3D structure of the protein.

Murrayanol and mahanimbine displayed similar binding affinity of −10.5 kcal/mol for eel AChE (1C2B). Murrayafoline A showed binding affinity of −7.7 kcal/mol for 1C2B. 9-Methyl-9H-carbazole-2-carbaldehyde displayed binding affinity of −8.2 kcal/mol for 1C2B. Figure 3A–D show 2D images of the carbazole derivatives’ binding interactions with eel AChE (1C2B). Figure 3E shows the binding sites in the 3D structure of the protein. The protein 1C2B consists of chain A. As depicted in Figure 3E, murrayanol, mahanimbine, murrayafoline A, and 9-methyl-9H-carbazole-2-carbaldehyde bind at the same active site. We observed that pi–pi (π-π stacked) interactions are formed at TRP286 by all four carbazole derivatives, at TYR124 (murrayanol and murrayafoline A), and at TYR72 (murrayanol and 9-methyl-9H-carbazole-2-carbaldehyde). 9-Methyl-9H-carbazole-2-carbaldehyde forms a carbon H-bond at ARG296. Mahanimbine forms a conventional H-bond at SER293. A pi–sigma (π-σ) bond at TRP286 is formed by mahanimbine and murrayafoline A. π-Alkyl bonds are formed by mahanimbine at LEU289, TYR124, TRP286, and PHE297 and by murrayafoline A at TYR72. Van der Waals interactions are also present: for murrayanol at TYR341, ARG296, LEU289, and HIS287; for mahanimbine at GLN291, GLU292, ARG296, ILE294, and PHE295; for murrayafoline A at PHE338, TYR337, TYR341, PHE295/297, ILE294, and ARG296; and for 9-methyl-9H-carbazole-2-carbaldehyde at TYR124/341, PHE297/295, LEU289, SER293, and ILE294.

#### 2.3.2. Docking with Aβ Receptors

Table 5 displays the binding affinity of the ligands for the octamer, pentamer, and monomer structures of the Aβ peptide. Among the analyzed compounds, murrayanol revealed a stronger binding with 2BEG (−8.0 kcal/mol) than with 8EZE (−7.4kcal/mol) and 1IYT (−6.2 kcal/mol), indicating an increased stable interaction with 2BEG. Similarly, murrayafoline A showed strong binding affinity for 2BEG (−7.0 kcal/mol), slightly less affinity for 8EZE (−6.5 kcal/mol), and the least binding affinity for 1IYT (−5.2 kcal/mol). Mahanimbine and 9-methyl-9H-carbazole-2-carbaldehyde, however, exhibited an increase in binding affinity (monomer < pentamer < octamer). Mahanimbine showed binding affinities of −8.0 kcal/mol for 8EZE, −7.6 kcal/mol for 2BEG, and −6.6 kcal/mol for 1IYT. Similarly, binding energies of −6.4 kcal/mol (8EZE), −5.9 kcal/mol (2BEG), and −5.1 kcal/mol (1IYT) were observed for 9-methyl-9H-carbazole-2-carbaldehyde. 

The binding affinity (kcal/mol) values of the carbazole derivatives are represented in Table 5.

**Figure 4:**
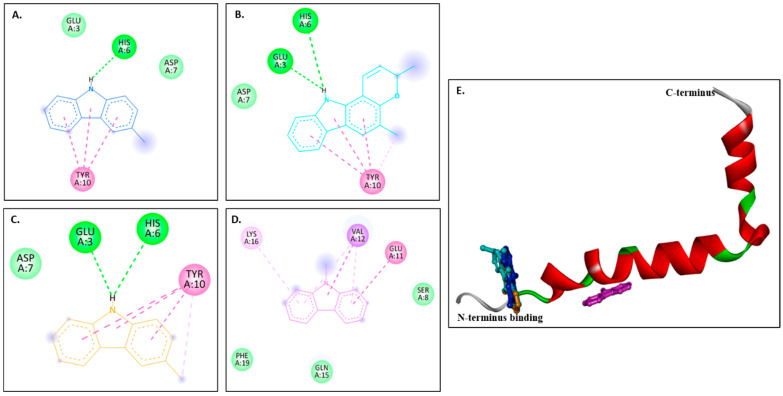
Visual representation of the interaction between ligands and receptor molecule (1IYT). Two-dimensional visualization of the binding interactions of (**A**) murrayanol (dark blue), (**B**) mahanimbine (sky blue), (**C**) murrayafoline A (orange), and (**D**) 9-methyl-9H-carbazole-2-carbaldehyde (pink). (**E**) Binding sites of the ligands in the 3D structure of the protein.

Figure 4A–D show 2D images of the binding interactions of the carbazole derivatives (ligands) with 1IYT. Figure 4E shows the binding sites in the 3D protein structure. Figure S4 displays the 3D binding interactions of the ligand molecules with the receptor. Murrayanol forms H-bonding with HIS6, and mahanimbine and murrayafoline A form H-bonding with HIS6 and GLU3 (Figure 4E). All three compounds also form a π-π (stacked) interaction with TYR10, and mahanimbine and murrayafoline A form a π–alkyl bond. 9-Methyl-9H-carbazole-2-carbaldehyde binds to the receptor molecule at a different site, forming a π–alkyl bond at LYS16 and VAL12, a π–amide bond at GLU11, and a π-σ bond at VAL12. Van der Waals interactions are also formed between the ligands and the receptor molecule: murrayanol at GLU3 and ASP7; mahanimbine and murrayafoline A at ASP7; and 9-methyl-9H-carbazole-2-carbaldehyde at SER8, PHE19, and GLN15.

**Figure 5:**
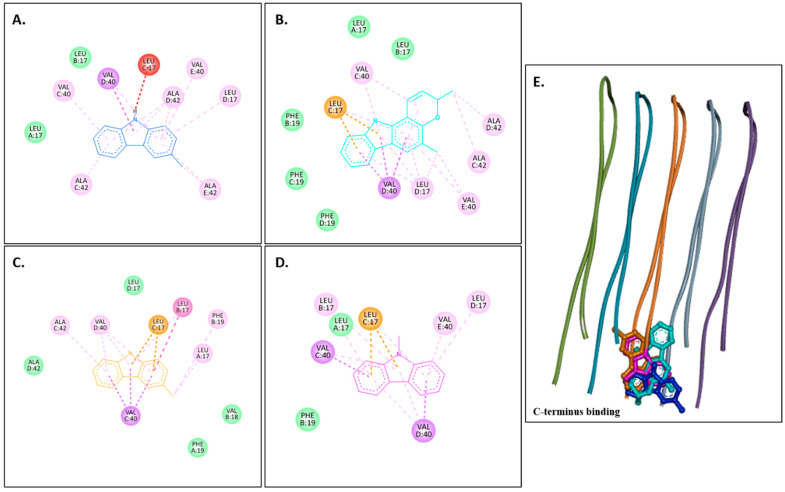
Visual representation of the interaction between ligands and receptor molecule (2BEG). Two-dimensional visualization of the binding interactions of (**A**) murrayanol (dark blue), (**B**) mahanimbine (sky blue), (**C**) murrayafoline A (orange), and (**D**) 9-methyl-9H-carbazole-2-carbaldehyde (pink). (**E**) Binding sites of the ligands in the 3D structure of the protein.

Figure 5A–D show 2D images of the carbazole derivatives (ligands)’ binding interaction with 2BEG. Figure 5E shows the binding sites in the 3D protein structure. Figure S5 displays the 3D binding interaction of the ligand molecules with the receptor. 2BEG consists of five chains, and all compounds bind at the same site. As shown, π-σ interactions were observed at VAL40 in the D chain for murrayanol and mahanimbine, VAL40 in the C chain for murrayafoline A, and VAL40 in the C/D chains for 9-methyl-9H-carbazole-2-carbaldehyde. π–Cation interactions were observed at LEU17 in the C chain for mahanimbine, murrayafoline A, and 9-methyl-9H-carbazole-2-carbaldehyde. π–Alkyl interactions were observed for murrayanol at VAL40 in the C/D/E chains, ALA42 in the C/D/E chains, and LEU17 in the D chain; for mahanimbine, at VAL40 in the C/D/E chains and LEU17 in the C/D chains; for murrayafoline A, at VAL40 in the D chain, ALA42 in the C chain, LEU17 in the B chain, and PHE19 in the B chain; and for 9-methyl-9H-carbazole-2-carbaldehyde, at VAL40 in the C/D/E chains and LEU17 in the B/C/D chains. π–Amide interactions were observed for murrayafoline A at LEU17 in the B chain. Alkyl interactions are formed by murrayanol at ALA42 in the E chain; mahanimbine at ALA42 in the C/D chains, VAL40 in the E chain, and LEU17 in the D chain; and murrayafoline-A at LEU17 in the A chain. Murrayanol N-H interacts with LEU17 in the C chain, forming an unfavorable donor–donor interaction that may reduce the stability of the protein–ligand complex because of the repulsion between their electron-rich H atoms. Van der Waals interactions are also present: for murrayanol at LEU17; for mahanimbine at LEU17, and PHE19; for murrayafoline A at ALA42, LEU17, PHE19, and VAL18; and for 9-methyl-9H-carbazole-2-carbaldehyde at LEU17, and PHE19.

**Figure 6:**
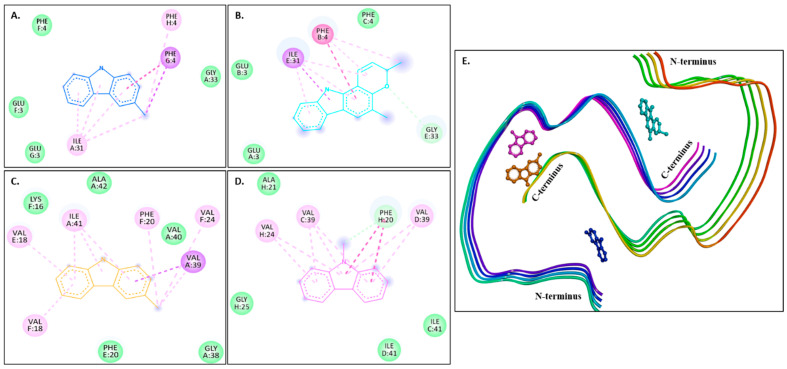
Visual representation of the interaction between ligands and receptor molecule (8EZE). Two-dimensional visualization of the binding interactions of (**A**) murrayanol (dark blue), (**B**) mahanimbine (sky blue), (**C**) murrayafoline A (orange), and (**D**) 9-methyl-9H-carbazole-2-carbaldehyde (pink). (**E**) Binding sites of the ligands in the 3D structure of the protein.

Figure 6A–D show 2D images of the carbazole derivatives (ligands)’ binding interaction with 8EZE. Figure 6E shows the binding sites in the 3D protein structure. Figure S6 displays the 3D binding interactions of the ligand molecules with the receptor. Interestingly, 8EZE consists of eight chains, and all compounds bind to all its different binding sites. π-π (stacked) interactions are formed by murrayanol at PHE4 in the G-chain and mahanimbine at PHE4 in the B-chain, and a π-π (T-shaped) interaction is formed by 9-methyl-9H-carbazole-2-carbaldehyde at PHE20 in the H-chain. A π-σ interaction is formed by murrayanol at PHE4 in the G-chain, mahanimbine at ILE31 in the E-chain, and murrayafoline A at VAL39 in the A-chain. π-Alkyl interactions are also formed by murrayanol at PHE4 in the H-chain and ILE31 in the A-chain, mahanimbine at ILE31 in the E-chain and PHE4 in the B-chain, murrayafoline A at VAL18 in the E/F chain, PHE20 in the F-chain, and ILE41 in the A-chain, and by 9-methyl-9H-carbazole-2-carbaldehyde at VAL24 in the H-chain and VAL39 in the C/D chains. Alkyl interactions were observed for murrayanol at ILE31 in the A-chain, for mahanimbine at ILE31 in the E-chain and PHE4 in the B-chain, and for murrayafoline A at VAL24 in the F-chain and VAL39 in the A-chain. Mahanimbine forms a carbon H-bond at GLY33 in the E-chain with the oxygen atom. A carbon H-bond is also formed by 9-methyl-9H-carbazole-2-carbaldehyde at PHE20 in the H-chain. van der Waals interactions were also observed: for murrayanol at PHE4 in the F-chain, GLU3 in the F/G chain, GLY33 in the A-chain; for mahanimbine at PHE4 in the C-chain, GLU3 in the A/B chain, GLY33 in the E-chain; for murrayafoline A at ALA42 in the A-chain, GLY38 in the A-chain, LYS16 in the F-chain, PHE20 in the E-chain, VAL40 in the A-chain; and for 9-methyl-9H-carbazole-2-carbaldehyde at ALA21 in the H-chain, GLY25 in the H-chain, ILE41 in the C/D chains.

### 2.4. In Vitro Acetylcholinesterase (AChE) Inhibitory Activity

AChE (E.C.3.1.1.7) is a cholinergic enzyme generally present at postsynaptic neuromuscular junctions and hydrolyses acetylcholine (ACh), an important neurotransmitter. In Alzheimer’s disease (AD) patients, the levels of ACh are reduced in synaptic junctions; hence, inhibition of AChE is desirable to maintain the normal ACh levels. Therefore, the extracts and the phytocompounds were screened for anti-AChE activity, and the IC_50_ values (half-maximal inhibitory concentration) were calculated using galantamine hydrobromide as an inhibitory control. 

**Figure 7:**
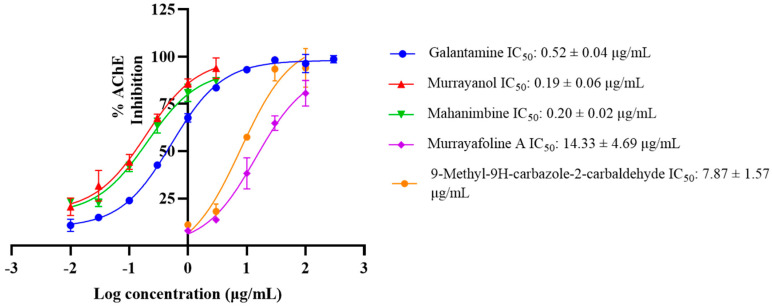
IC_50_ curves for murrayanol, mahanimbine, murrayafoline A, and 9-methyl-9H-carbazole-2-carbaldehyde against *Electrophorus electricus* AChE. Galantamine was used as a positive control. The IC_50_ values were calculated using GraphPad Prism 10.3. The values were determined as the mean from three experiments ± SD.

Murrayanol and mahanimbine displayed higher (IC_50_: 0.19 ± 0.06 μg/mL and 0.20 ± 0.02 μg/mL, respectively) and better anti-AChE activity in comparison to the positive control. The IC_50_ value of galantamine was 0.52 ± 0.04 μg/mL, similar to the previously reported value of 0.31 μg/mL [8], while 9-methyl-9H-carbazole-2-carbaldehyde (IC_50_: 7.87 ± 1.57 μg/mL) and murrayafoline A showed lower anti-AChE activity (IC_50_: 14.33 ± 4.69 μg/mL) in comparison to the other compounds (Figure 7).

**Figure 8:**
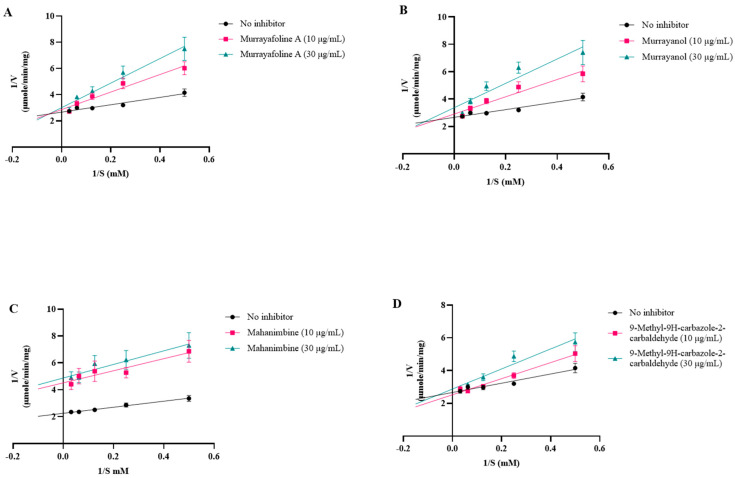
Inhibition plots (Lineweaver–Burk plots) for (**A**) murrayafoline A; (**B**) murrayanol; (**C**) mahanimbine; and (**D**) 9-methyl-9H-carbazole-2-carbaldehyde.

The inhibition curves were plotted using linear regression (Lineweaver–Burk plot) on GraphPad Prism 10.3 (Figure 8).

The K_m_ and V_max_ values were calculated using a non-linear fit (Michaelis–Menten equation) (Table 6). The K_i_ value (inhibitor constant), the dissociation constant describing the binding affinity between the inhibitor and the enzyme, was calculated as described previously [9,10].

Competitive inhibition patterns were observed for murrayanol, murrayafoline A, and 9-methyl-9H-carbazole-2-carbaldehyde, indicating their competitive inhibition with the substrate at the enzyme’s active site. On the other hand, mahanimbine displayed non-competitive inhibition, indicating that it may bind to the enzyme elsewhere on the protein. Murrayanol had the lowest K_i_ value (~0.45 μM) in comparison to the other compounds, showing the strongest binding affinity for the enzyme, followed by mahanimbine (0.59 μM), 9-methyl-9H-carbazole-2-carbaldehyde (~30 μM), and murrayafoline A (~60 μM).

### 2.5. Anti-Aβ Fibrillization Activity

The compounds’ Aβ fibrillization inhibitory activity was analyzed by the thioflavin (ThT) assay. Increased ThT binding to the targeted β-sheet of amyloid fibrils could be assessed by monitoring fluorescence during fibril formation. The process of ThT attachment to β-fibrils requires structural compatibility with the fibrils’ voids, is characterized by increased fluorescence following attachment, and is subjected to the effects of environmental elements, like ionic strength and polypeptide flexibility [11]. 

**Figure 9:**
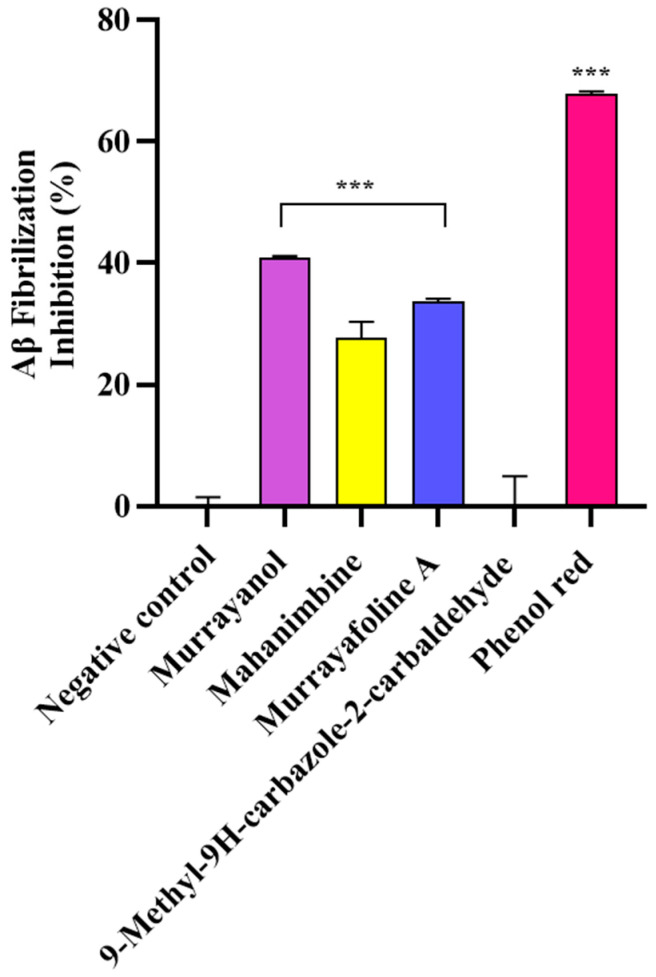
Aβ fibrillization inhibition by *Murraya* carbazole derivatives at 500 μg/mL. Phenol red (50 μM) was used as a positive control. All data are expressed as mean ± SD (*n* = 3). A significant difference *** (*p* < 0.001) using one-way ANOVA followed by Dunnett’s post hoc was observed in the percent oligomerization reduction vs. that of the negative control (buffer + Aβ).

In the study, the samples (500 μg/mL) were screened for their anti-fibrillization potential using phenol red as a positive control. The results were statistically significant (*** *p* < 0.001) in comparison to those obtained for the negative control (buffer + Aβ) (Figure 9). Murrayanol exhibited potent inhibition (40.83 ± 0.30%), followed by murrayafoline A (33.60 ± 0.55%) and mahanimbine (27.68 ± 2.71%). 9-Methyl-9H-carbazole-2-carbaldehyde did not show any inhibition. The inhibition by the positive control, phenol red (67.86 ± 0.38%), was similar to a previously reported value [12].

## 3. Discussion

The present study investigated the neuroprotective potential of selected *Murraya* carbazole derivatives using both in vitro and in silico approaches. Specifically, their ability to inhibit AChE and Aβ fibrillization in vitro was evaluated. 

SwissADME predicted the interactions of the compounds with important CYP enzymes, namely, CYP1A2, CYP2C19, CYP2C9, CYP2D6, and CYP3A4. CYP450 enzymes are critical for metabolizing drugs and facilitating their transport across cell membranes. While over 50 CYP enzymes exist, 6 are responsible for metabolizing approximately 90% of all drugs [13], with CYP3A4 and CYP2D6 being the two most significant ones. Other important CYPs include CYP2C19 (~15%) [14], CYP1A2 (~10%) [15], and CYP2C9 (~5%) [16]. Inhibition of these CYP enzymes could lead to drug toxicity due to drug–drug interactions, as a drug inhibiting a CYP enzyme reduces the enzyme’s ability to metabolize other drugs, potentially increasing their concentration and leading to adverse effects or toxicity.

In our study, 9-methyl-9H-carbazole-2-carbaldehyde displayed a favorable profile, as it did not inhibit the two major CYPs (CYP3A4 and CYP2D6) or CYP2C9. Similarly, murrayanol did not inhibit CYP2C9, CYP3A4, and CYP1A2, while murrayafoline A did not inhibit CYP2C9. These findings suggest that murrayanol and 9-methyl-9H-carbazole-2-carbaldehyde present a lower risk of CYP-mediated drug–drug interactions than the other compounds tested. 9-Methyl-9H-carbazole-2-carbaldehyde exhibited the broadest non-inhibitory profile, which is particularly significant, given the central role of CYP3A4 and CYP2D6 in drug metabolism.

According to SwissADME predictions, both murrayafoline A and 9-methyl-9H-carbazole-2-carbaldehyde can cross the blood–brain barrier (BBB), a crucial attribute for potential neuroprotective agents. Another significant advantage for a drug candidate is not being a P-gp substrate. P-gp, a transporter protein found in the liver, kidneys, intestines, and, notably, at the BBB, actively pumps foreign substances, including many drugs, out of the brain and other organs. Therefore, compounds not recognized by P-gp are likely to achieve and maintain better brain retention, which makes them more promising for neurological applications.

In this context, murrayanol and 9-methyl-9H-carbazole-2-carbaldehyde were predicted not to be P-gp substrates, which is encouraging. Although murrayanol possesses strong in vitro and docking activity, its anticipated poor permeability across the BBB will be a limiting factor for its bioavailability in the CNS. This can be circumvented via structural variation (e.g., prodrug design, nanoparticle delivery) or co-formulation approaches that enhance BBB penetration. Therefore, although its inherent neuroprotective activity is encouraging, optimization of its pharmacokinetics for its future development is required.

Conversely, murrayafoline A can cross the BBB but was predicted to be a P-gp substrate, meaning that it may enter the brain, but could be actively pumped out, which would reduce its effective concentration in the brain tissue.

Among the tested compounds, 9-methyl-9H-carbazole-2-carbaldehyde stood out as the most promising, based on its favorable ADME/T properties, being both BBB-permeable and not a P-gp substrate. This suggests its potential to achieve and maintain therapeutically relevant concentrations in the brain. However, further in-depth study is required to fully understand the molecular mechanisms of amyloidogenesis and to facilitate the structure-based development of anti-Aβ drugs.

To gain a deeper understanding of ligand–protein interactions, molecular docking studies were performed, and the compounds’ physicochemical parameters, pharmacokinetics, drug-likeness, and toxicity were predicted.

The docking results for human AChE (PDB: 4EY7) showed that murrayanol, mahanimbine, and 9-methyl-9H-carbazole-2-carbaldehyde can bind to chain A, while murrayafoline A can bind to chain B of the receptor protein. Mahanimbine exhibited the strongest binding affinity (−12.4 kcal/mol), followed by murrayanol (−11 kcal/mol), murrayafoline A (−9.1 kcal/mol), and 9-methyl-9H-carbazole-2-carbaldehyde (−8.8 kcal/mol). As indicated in Table 4, the binding affinities of the carbazole derivatives for *Electrophorus electricus* AChE (PDB: 1C2B) were weaker than those for human AChE (PDB: 4EY7). This finding aligns with our in vitro results, as murrayanol and mahanimbine exhibited the lowest Ki values (0.45 μM and 0.59 μM), indicating the strongest binding affinity for the enzyme. A sequence alignment between human AChE (UniProt ID: P22303) and *Electrophorus electricus* AChE (UniProt ID: P21836) was performed using CLUSTAL ver. 1.2.4 on the UniProt web server, revealing an 88.44% identity matrix between the two sequences.

For the Aβ docking studies, three different forms of Aβ, i.e., monomer, pentamer, and octamer, were used. The docking results for the Aβ monomer (PDB: 1IYT) indicated that ligand binding occurred primarily at the N-terminus of the peptide. Murrayanol, mahanimbine, and murrayafoline A form conventional hydrogen bonds with the Aβ monomer, which could influence the structural integrity and biological function of these compounds. The N-terminus domain is typically associated with the structure and toxicity of Aβ oligomers [17]. It is evident that the N-terminus of Aβ plays a crucial role in amyloid fibrillization, leading to the formation of secondary structures and ultimately neuronal death [17]. Therefore, targeting the N-terminus could prove highly beneficial therapeutically, suggesting that these carbazole derivatives may minimize fibril/oligomer formation by binding to the N-terminal of the Aβ monomer.

In the case of the Aβ pentamer (PDB: 2BEG), residues 1–17 are disordered, while residues 18–42 are responsible for forming β-sheets [18]. In our study, all ligand–receptor binding occurred at the C-terminus of the Aβ peptide, a region critical for protein oligomerization [19]. Interestingly, in regard to the Aβ octamer (PDB: 8EZE), murrayanol and mahanimbine docked at both the N- and the C-termini of the peptide, albeit at different positions, suggesting that both compounds could help reduce fibril and oligomer formation. Murrayafoline A (A-chain at VAL39, ILE41) and 9-methyl-9H-carbazole-2-carbaldehyde (C and D-chains at VAL39) bound at the C-terminus of the peptide. Considering the binding sites of these ligand molecules, it could be inferred that these compounds target the Aβ42 peptide at precise positions critical for inhibiting Aβ fibrillization and oligomerization.

Deficiency in ACh in the cerebral cortex is a major feature in AD. The cholinergic hypothesis states that memory deficits in senile dementia arise from a specific and permanent lack of cholinergic activity in the brain [20]. While AChE normally modulates ACh by facilitating its degradation, excessive AChE activity leads to constant ACh deficiency, severely impairing memory and other cognitive abilities in affected individuals. AChE inhibitors play a pivotal role in delaying the progression of neurodegenerative diseases by preserving cholinergic signaling and slowing degenerative changes. Researchers are investigating the potential of plants, especially those with AChE inhibitory activity, to slow down neurodegeneration, particularly in diseases like AD [21,22].

In our study, all tested carbazole derivatives inhibited AChE activity. For our study, AChE from the electric eel (*E. electricus*) was used as it shares structural similarity with human AChE, particularly in its active sites. This similarity allows for reliable modeling of AChE inhibition, particularly in preliminary screening studies. Its structure (PDB ID: 1C2B) has been previously resolved in complex with inhibitors such as tacrine, making it a relevant model for anti-Alzheimer drug screening in neuropharmacological research [23]. Murrayanol and mahanimbine demonstrated particularly potent inhibition, with similar IC_50_ values (~0.2 μg/mL), and were more effective than the positive control, galantamine (IC_50_ value 0.52 ± 0.04 μg/mL). The IC_50_ values of 9-methyl-9H-carbazole-2-carbaldehyde and murrayafoline A were 7.8 ± 1.57 μg/mL and 14.3 ± 4.69 μg/mL, respectively. A previous study reported that the IC_50_ value of mahanimbine was 0.03 ± 0.09 mg/mL against AChE from bovine erythrocytes [24]. The disparity in the IC_50_ values, with our observed values being lower (corresponding to more potent inhibitors) than that of mahanimbine, could be attributed to the use of different sources for AChE (bovine erythrocytes or *E. electricus*). Prior research also showed that mahanimbine pre-treatment improved the ACh levels and inhibited AChE in the brain of LPS-treated animal models [25]. To date, no literature reports the AChE inhibitory activity of the other three carbazole derivatives. In the current study, all carbazole derivatives inhibited AChE through both competitive and non-competitive mechanisms, to a varying extent influenced by their structural characteristics. Non-competitive inhibitors usually possess extended side chains that can alter enzyme conformations via binding at allosteric sites. Mahanimbine’s non-competitive nature could be attributed to its bulky hydrophobic profile. Based on the docking results, mahanimbine binds to the peripheral anionic site (PAS) of the AChE protein (4EY7). This result could further be validated by using tacrine, which is a known non-competitive inhibitor binding at the PAS site of AChE (Figure S7). Murrayanol and mahanimbine exhibited robust binding affinities (Ki 0.44–0.59 μM) for AChE, suggesting their potential as potent AChE inhibitors to enhance cholinergic signals within biological systems.

In the ThT assay, significant Aβ-fibrillization inhibition was observed by murrayanol (40.83 ± 0.30%), murrayafoline A (33.60 ± 0.55%), and mahanimbine (27.68 ± 2.71%). In contrast, 9-methyl-9H-carbazole-2-carbaldehyde showed no inhibition. ThT binds to regions rich in aromatic and hydrophobic residues within the parallel β-sheet of fibrils [26]. The in vitro results suggest that 9-methyl-9H-carbazole-2-carbaldehyde might bind away from these β-sheet grooves, thus exhibiting docking affinity but no anti-fibrillization activity in the ThT assay. This discrepancy highlights a key limitation of molecular docking, which provides a theoretical model of ligand–protein interactions but does not account for the full complexity of the in vitro or in vivo biological environment. The varied inhibitory effects of the carbazole derivatives on Aβ fibrillization could be comprehensively analyzed by considering their structural characteristics, physicochemical traits, and binding mechanisms. Murrayanol and murrayafoline A possess functional groups that facilitate significant interactions with Aβ peptides. These functional groups enable robust non-covalent interactions, including hydrogen bonding, π-π stacking, and hydrophobic interactions, with Aβ fibrils. Such interactions would be crucial for influencing the binding of drug compounds to their target proteins and stabilizing protein 3D structures. The π-π stacking interaction, formed between the aromatic ring of the ligand and an aromatic residue of the protein, enhances the binding affinity and increases the docking score due to strong non-covalent contributions [27]. In contrast, π-π T-shaped interactions, formed when an aromatic ring interacts perpendicularly with the π-cloud of another aromatic ring, are considered weaker and often occur at peripheral or hydrophobic sites [28,29]. The main structural features of murrayanol that contributes to preventing amyloid fibrillization include its tricyclic carbazole backbone, hydroxyl groups, aromatic rings for π-π stacking interactions, and a hydrophobic prenyl chain. The methoxy group in murrayafoline A may influence its interactions with amyloid proteins by facilitating hydrophobic and hydrogen bonding, which are vital in disrupting the aggregation pathway [30]. Due to their structural adaptability, these functional groups effectively influence the aggregation processes and prevent Aβ fibrillization [31].

Amyloid plaques, primarily composed of Aβ peptides of 40~42 amino acids, accumulate into fibrils with a high β-sheet structure and are a typical pathological feature of AD. Amyloid fibrils are known to interact with cellular receptors, disrupt signal transduction pathways, increase reactive oxygen species (ROS), trigger inflammation, activate the immune system, and ultimately cause cell death [32]. Therefore, compounds capable of inhibiting fibrillization hold significant promise for treating AD. The challenges in developing drugs for amyloid-related diseases stem from structural constraints, inadequate binding affinity for amyloid proteins, and complex interaction dynamics that hinder amyloid fibril formation. Even though previous work indicated that mahanimbine reduced Aβ1-40 levels, but not Aβ1-42, it still improved cognition in an animal model [25]. This specificity could be attributed to structural variances, differing binding dynamics, and various biological factors influencing their interactions. Furthermore, murrayafoline A demonstrated neuroprotective activity in microglial cells by reducing inflammation [33], a process crucial in the development of amyloid-related pathologies in neurodegenerative diseases.

## 4. Materials and Methods

### 4.1. Chemicals 

Acetylcholinesterase (*Electrophorus electricus*, Type VI-S), acetyl thiocholine chloride (ATC), 5,5′-dithiobis-(2-nitrobenzoic acid) (DTNB), galantamine, thioflavin T (ThT), phenol red, murrayafoline A, and 9-methyl-9H-carbazole-2-carbaldehyde were from Sigma-Aldrich (St. Louis City, MO, USA)**,** and Aβ_1–42_ was purchased from GenicBio Inc. (Shanghai, China). Murrayanol was ordered from Aobious (Gloucester, MA, USA)**,** and mahanimbine was provided by Toronto Research Chemicals Inc. (North York, ON, Canada).

### 4.2. In Silico Studies

#### 4.2.1. Ligand Structure Selection

The following ligands were downloaded from PubChem (https://pubchem.ncbi.nlm.nih.gov/, accessed on 8 March 2025): Pub ID: 9975970, murrayanol; Pub ID: 167963, mahanimbine; Pub ID: 375150, murrayafoline A; and Pub ID: 3152662, 9-methyl-9H-carbazole-2-carbaldehyde. 

#### 4.2.2. Protein Structure Selection and Preparation

The structural receptors for AChE (PDB ID: 4EY7, 1C2B) and Aβ_1-42_ (PDB IDs: 1IYT, 2BEG, and 8EZE) were downloaded from the Protein Data Bank (PDB). Aβ has a very disordered conformation and high flexibility. These properties enable Aβ to exist in multiple conformations, including monomeric, oligomeric, β-sheet, and fibril forms, which are highly toxic. The purpose of using three different conformations of the same peptide, i.e., 1IYT (monomer), 2BEG (helical structure), and 8EZE (fibril), was to evaluate the binding of the ligand molecules across multiple forms of the protein. This would also help to evaluate and identify ligands with broad anti-Aβ activity. The UCSF ChimeraX program was used to prepare the protein structures for docking. Since the deposited structures may be unsuitable for direct usage, the Dock Prep tool was used to add any missing atoms, H-bonds, and a partial charge to the protein structure [34].

#### 4.2.3. Drug-Likeness, Toxicity Predictions, and ADME Properties of the Compounds

The compounds’ drug-likeness based on their physicochemical properties, pharmacokinetics, bioavailability, and ADME parameters (absorption, distribution, metabolism, and excretion) was predicted using SwissADME (http://www.swissadme.ch/index.php, accessed on 14 April 2025), a freely accessible web tool. Toxicity predictions for the compounds were performed using the ProTox3.0 web tool (https://tox.charite.de/protox3/index.php, accessed on 14 April 2025).

#### 4.2.4. Molecular Docking

Molecular docking is an important step in the drug design and drug discovery process. To better understand how the four carbazole derivatives bind to the examined receptor proteins, molecular docking was performed using the PyRx 0.8 program. It consists of AutoDock Wizard, Vina Wizard, and Open Babel built-in programs, which make it quite easy to access. Energy minimization of the compounds was performed, and conversion to the PDBQT format was achieved using the AutoDock Ligand format in PyRx. Biovia Discovery Studio (BDS) was used for viewing the 3D structures of protein–ligand complexes [35]. Conformations with the least binding energies are considered more favorable for analyzing and predicting interactions between receptors and ligands.

### 4.3. Anti-Acetylcholinesterase (AChE) Activity

AChE inhibition was measured using Ellman’s method with slight modifications [36]. The compounds were incubated with the enzyme and the substrate (10 mM ATC) for 15 min. Later, 15 mM DTNB was added, and the plate was incubated for 10 min at 37 °C. The absorbance was measured at 412 nm using a multimode reader (Synergy-H1 BioTek, Agilent, Santa Clara, CA, USA). Galantamine served as a positive control. Percent inhibition was computed using the equation:

Inhibition percentage (I%) = [(A_1_ − A_2_) − (A_3_ − A_4_)]/(A_1_ − A_2_) × 100, where A_1_ = absorbance without inhibitor; A_2_ = negative control; A_3_ = absorbance of inhibitor; and A_4_ = negative control with inhibitor.

### 4.4. Thioflavin T (ThT) Assay

The anti-Aβ_1-42_ fibrilization activity of the compounds was measured using the ThT assay, as previously reported [12]. The compounds were incubated at 37 °C in the presence/absence of Aβ_1-42_. After 24 h of incubation, ThT (100 µM) was added, and the reaction was incubated at 37 °C for 15 min. Fluorescence was determined at Ex 450 nm/Ems 490 nm using a multimode reader (Victor3, PerkinElmer, Shelton, CT, USA). Phenol red (50 µM) was used as a positive control, and percent fibrillization inhibition was computed as:

Percent Aβ_1-42_ fibrilization inhibition (I%) = [(1 − Fi/Fc) × 100]

where Fi = fluorescence intensity with inhibitor; Fc = fluorescence intensity without inhibitor.

### 4.5. Statistical Analysis

Statistical analysis was performed by one-way ANOVA followed by Dunnett’s post hoc test. The data were registered as the mean ± SD of at least three experiments. The symbol *** represent *p* < 0.001 significance compared to the control. The IC_50_ values were determined using non-linear regression, and the Lineweaver–Burk plots were drawn using linear regression analysis by GraphPad Prism 10.3.

## 5. Conclusions

This study comprehensively evaluated the neuroprotective activity of four Murraya carbazole derivatives through a series of integrated in vitro and in silico investigations. The findings collectively highlighted their multi-target potential relevant to AD therapy. AChE inhibition and binding affinity: All four compounds demonstrated significant in vitro inhibition of acetylcholinesterase (AChE), evidenced by their low IC_50_ values. Correspondingly, molecular docking studies revealed their strong binding affinities for human AChE receptor molecules, supporting the observed enzymatic inhibition.

Aβ42 interaction and anti-fibrillization potential: Docking simulations with Aβ42 demonstrated strong binding affinities for different oligomeric forms of the peptide. This interaction profile suggests that these compounds are likely to reduce Aβ fibrillization and oligomerization, key pathological events in AD progression.

Comprehensive analyses of drug-likeness, toxicity, and other pharmacokinetic properties (ADME/T) were also performed. Based on the ADME/T properties, 9-methyl-9H-carbazole-2-carbaldehyde showed particularly promising results. Its ability to cross the blood–brain barrier (BBB) and its non-substrate status for P-glycoprotein (P-gp) suggest a favorable profile for central nervous system (CNS) therapeutic applications. The results strongly suggest the therapeutic potential of these ligand molecules as inhibitors for AD. Nevertheless, more in-depth research will be crucial to fully elucidate the intricate mechanisms of molecular amyloidogenesis and to advance the structure-based development of novel anti-Aβ drugs.

## Figures and Tables

**Table 1:** Drug-likeness prediction for carbazole derivatives.

Compounds	LIPO (CLogP)	Size (g/mol)	Polar (TPSA)	INSOLU LogS (Esol)	UNSATU (Fsp3)	Flex (RB)	Lipinski’s Rule	Bioavailability Score
Murrayanol	5.71	363.49	45.25	−6.56	0.33	6	1	0.55
Mahanimbine	5.62	331.45	25.02	−6.26	0.3	3	1	0.55
Murrayafoline A	3.31	211.26	25.02	−4.03	0.14	1	0	0.55
9-Methyl-9H-carbazole-2-carbaldehyde	2.71	209.24	22	−3.44	0.07	1	0	0.55

Abbreviations: Flex: flexibility; Fsp3: fraction of sp3 carbon atoms; UNSATU: unsaturation; INSOLU: insolubility; LIPO: lipophilicity; RB: number of rotational bonds; TPSA: topological polar surface area.

**Table 2:** ADME properties of carbazole derivatives.

Compounds	CYP1A2	CYP2C19	CYP2C9	CYP2D6	CYP3A4	GI Absorption	BBB Permeability	P-gp Substrate
Murrayanol	No	Yes	No	Yes	No	High	No	No
Mahanimbine	Yes	Yes	Yes	Yes	Yes	High	No	Yes
Murrayafoline A	Yes	Yes	No	Yes	Yes	High	Yes	Yes
9-Methyl-9H-carbazole-2-carbaldehyde	Yes	Yes	No	No	No	High	Yes	No

Abbreviations: CYP: cytochrome P450 system; GI: gastrointestinal; BBB: blood–brain barrier; P-gp: P-glycoprotein.

**Table 3:** Toxicity analysis of the carbazole derivatives.

Compounds	Hepatotoxicity	Cytotoxicity	Neurotoxicity	LD_50_ (mg/kg)	Toxicity class
Murrayanol	Inactive	Inactive	Inactive	2300	V
Mahanimbine	Inactive	Inactive	Inactive	4000	V
Murrayafoline A	Inactive	Inactive	Active	1200	III
9-Methyl-9H-carbazole-2-carbaldehyde	Inactive	Inactive	Active	1250	IV

Based on ProTox3.0 prediction. Active: indicates a predicted toxic effect; Inactive: denotes a non-toxic effect. LD: Lethal dose.

**Table 4:** Binding affinities (kcal/mol) of ligand molecules for AChE receptor proteins.

Compounds	Binding Affinities (kcal/mol)	
	4EY7	1C2B
Murrayanol	−11	−10.5
Mahanimbine	−12.4	−10.5
Murrayafoline A	−9.1	−7.7
9-Methyl-9H-carbazole-2-carbaldehyde	−8.8	−8.2

**Table 5:** Binding affinities (kcal/mol) of the ligand molecules for Aβ receptor proteins.

Compounds	Binding Affinities (kcal/mol)		
	1IYT	2BEG	8EZE
Murrayanol	−6.2	−8.0	−7.4
Mahanimbine	−6.6	−7.6	−8.0
Murrayafoline A	−5.2	−7.0	−6.5
9-Methyl-9H-carbazole-2-carbaldehyde	−5.1	−5.9	−6.4

**Table 6:** Kinetic parameters for acetylcholinesterase inhibition by *Murraya* carbazole derivatives.

	V_max_ (μmole/min/mg)	K_m_ (mM)	K_i_ (µM)	Inhibition Type
No Inhibitor	0.3710	0.9674		
Murrayanol 10 μg/mL Murrayanol 30 μg/mL	0.3756 0.3613	3.011 4.894	0.444 0.470	Competitive
Mahanimbine 10 μg/mL Mahanimbine 30 μg/mL	0.2098 0.2231	1.150 0.9881	0.599 0.599	Non-Competitive
Murrayafoline A 10 μg/mL Murrayafoline A 30 μg/mL	0.3826 0.3753	3.227 4.505	58.71 61.04	Competitive
9-Methyl-9H-carbazole-2-carbaldehyde 10 μg/mL 9-Methyl-9H-carbazole-2-carbaldehyde 30 μg/mL	0.3874 0.3634	1.733 2.509	29.18 31.35	Competitive

## Data Availability

Data are contained within the article and Supplementary Materials.

## Supplementary Materials

The following supporting information can be downloaded at: https://www.mdpi.com/article/10.3390/molecules30153138/s1, Figure S1: Bioavailability radars of carbazole derivative compounds; Figure S2: Boiled egg plots of carbazole compounds; Figure S3: Binding interaction of ligands in 3D structure of the protein (4EY7); Figure S4: Binding interaction of ligands in 3D structure of the protein (1IYT); Figure S5: Binding interaction of ligands in 3D structure of the protein (2BEG); Figure S6: Binding interaction of ligands in 3D structure of the protein (8EZE); Figure S7: Binding interaction of mahanimbine and tacrine to AChE (7XN1).

## Abbreviations

The following abbreviations are used in this manuscript:

AChE	Acetylcholinesterase
ADMET	Absorption, distribution, metabolism, excretion and toxicity
Aβ	Amyloid β
BBB	Blood–brain barrier
CNS	Central nervous system
CYP	Cytochrome P450
FLEX	Flexibility
Fsp3	Fraction of sp3 carbon atoms
UNSATU	Unsaturation
INSOLU	Insolubility
LIPO	Lipophilicity
PAS	Peripheral anionic site
P-gp	P-glycoprotein
ROS	Reactive oxygen species
ThT	Thioflavin T
TPSA	Topological polar surface area
