# Supplementary material for: Neuroprotective Evaluation of Murraya Carbazoles: In Vitro and Docking Insights into Their Anti-AChE and Anti-Aβ Activities
Source: Molecules. 2025 Jul 26;30(15):3138. doi: 10.3390/molecules30153138 (PMC12348157; doi:10.3390/molecules30153138)

Supporting Information

Figure S1: Bioavailability radars of carbazole derivative compounds: Murrayanol, Mahanimbine, Murrayafoline-A and 9-methyl-9H-carbazole-2-carbaldehyde.

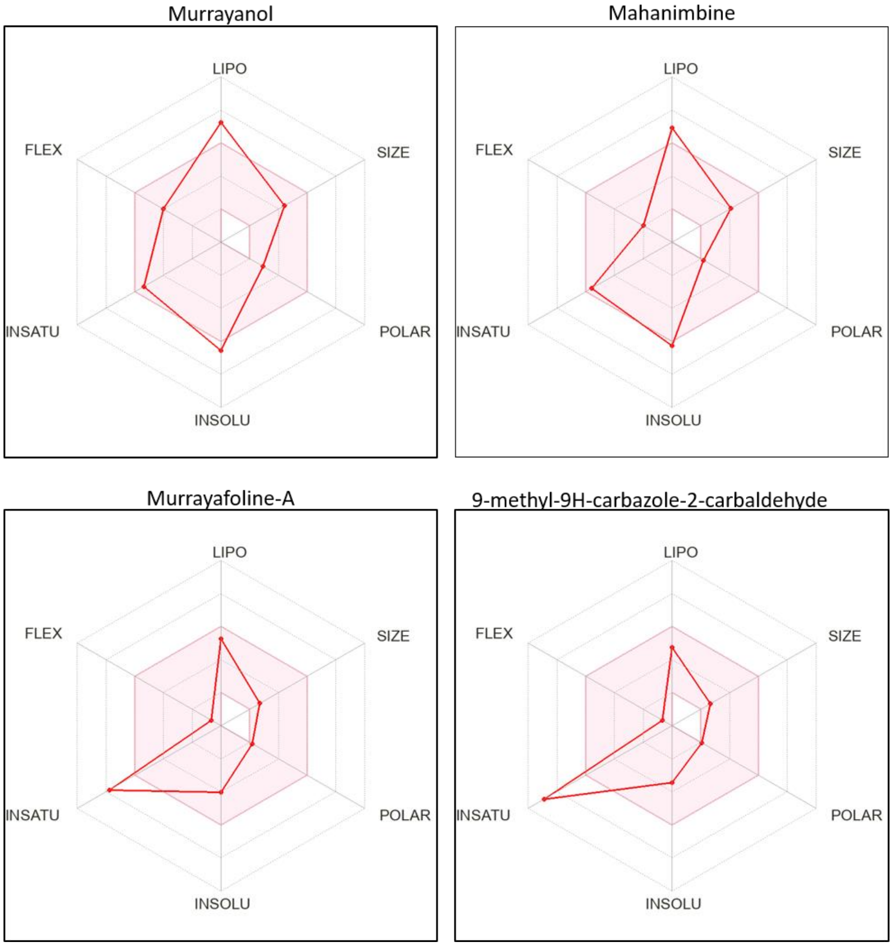

Figure S2: Boiled egg plots of carbazole compounds: Murrayanol, Mahanimbine, Murrayafoline-A and 9-methyl-9H-carbazole-2-carbaldehyde.

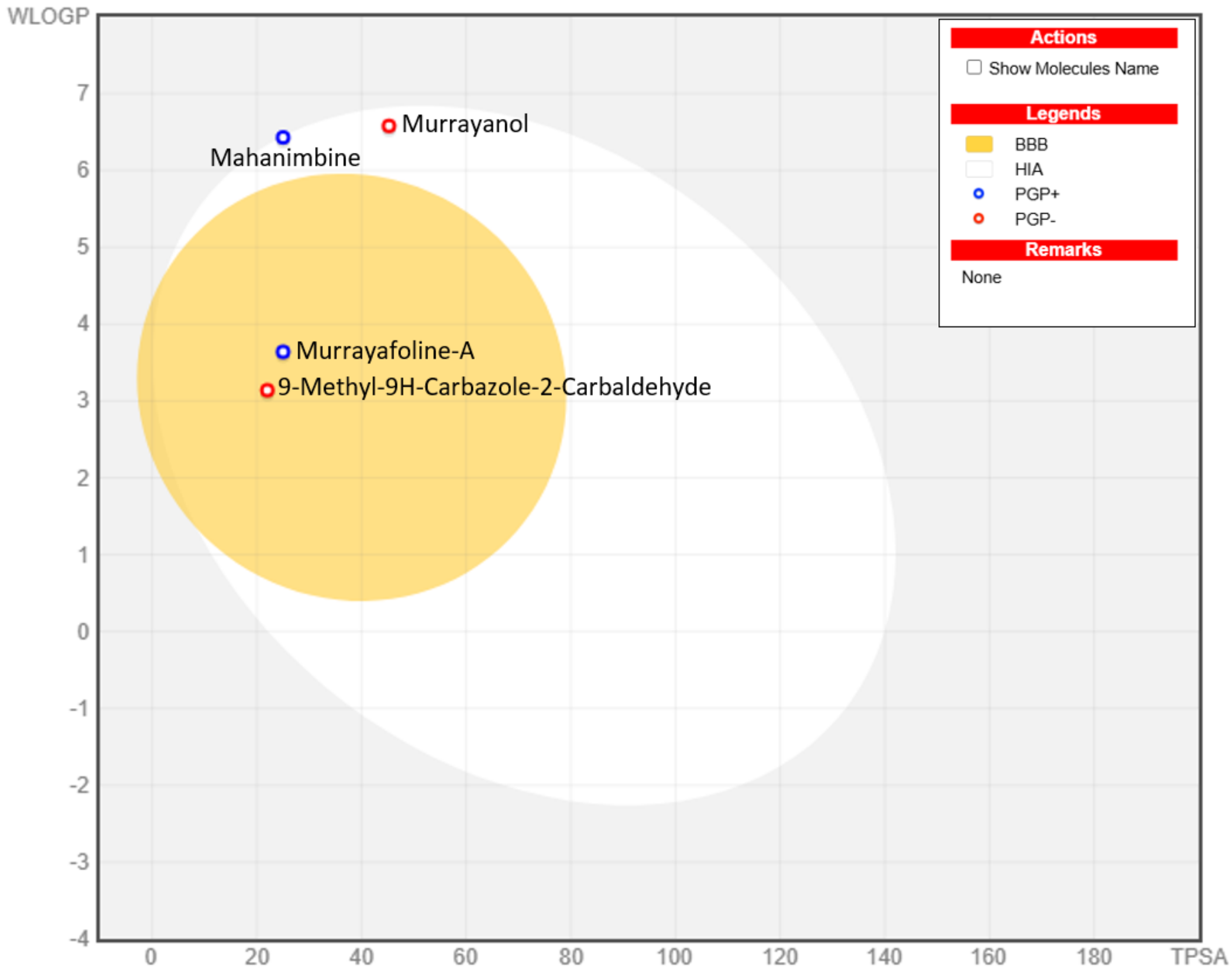

Figure S3: Binding interaction of ligands in 3D structure of the protein (4EY7). (A) murrayanol (dark blue), (B) mahanimbine (sky blue), (C) murrayafoline-A (orange), and (D) 9-methyl-9H-carbazole-2-carbaldehyde (pink).

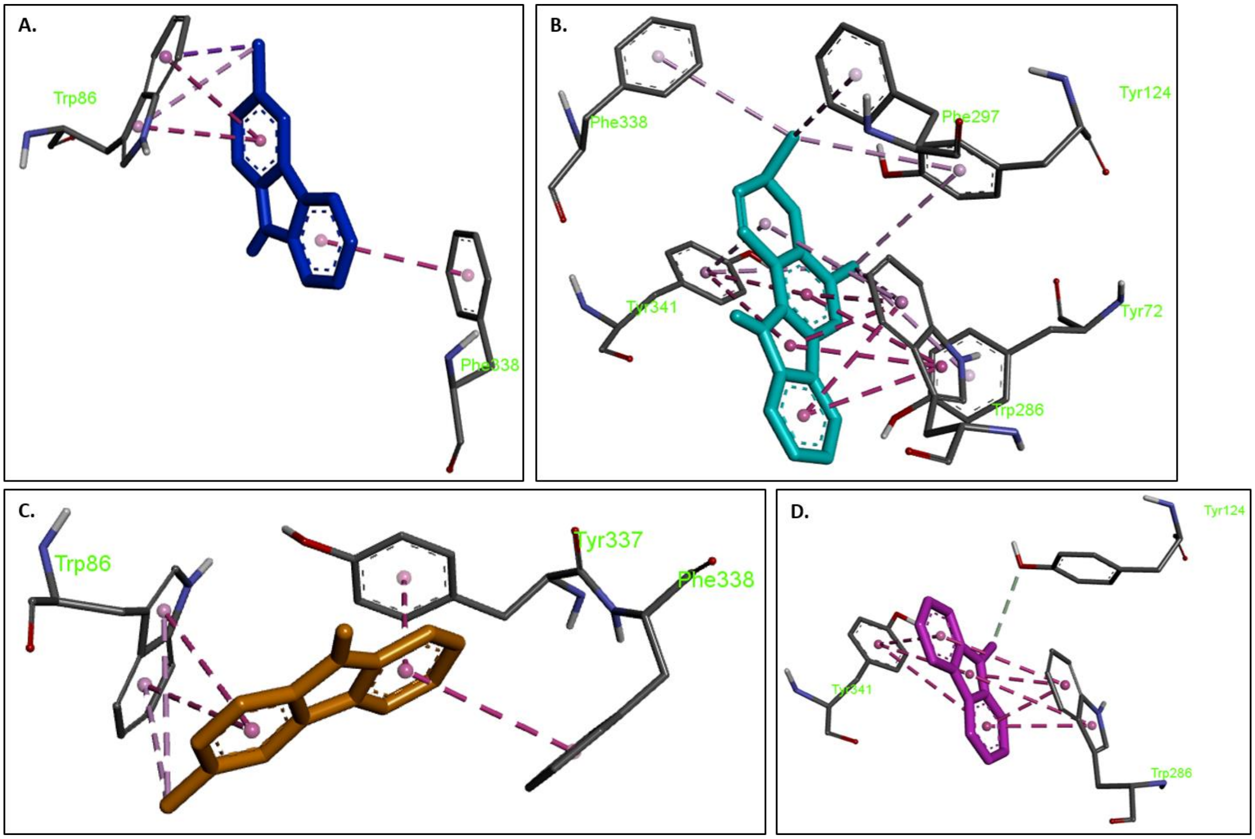

Figure S4: Binding interaction of ligands in the 3D structure of the protein (1IYT). (A) murrayanol (dark blue), (B) mahanimbine (sky blue), (C) murrayafoline-A (orange), and (D) 9-methyl-9H-carbazole-2-carbaldehyde (pink).

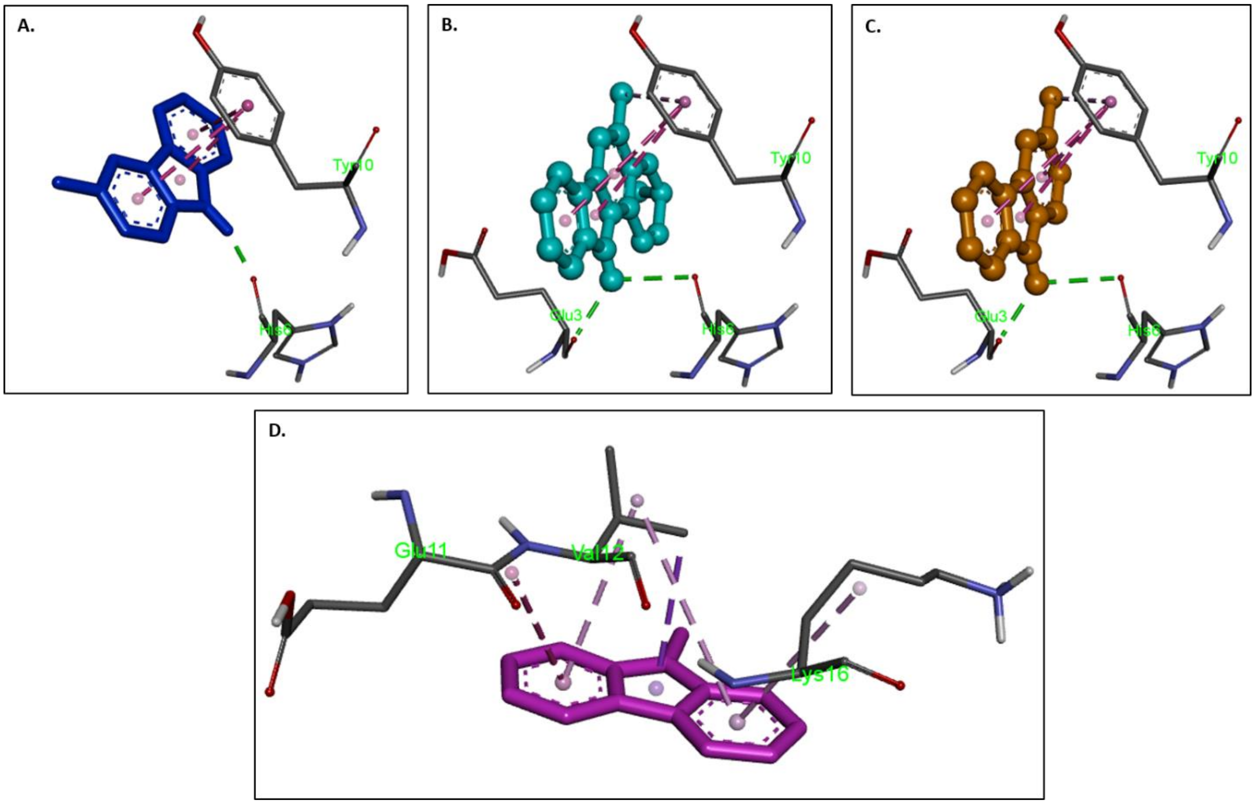

Figure S5: Binding interaction of ligands in 3D structure of the protein (2BEG). (A) murrayanol (dark blue), (B) mahanimbine (sky blue), (C) murrayafoline-A (orange), and (D) 9-methyl-9H-carbazole-2-carbaldehyde (pink).

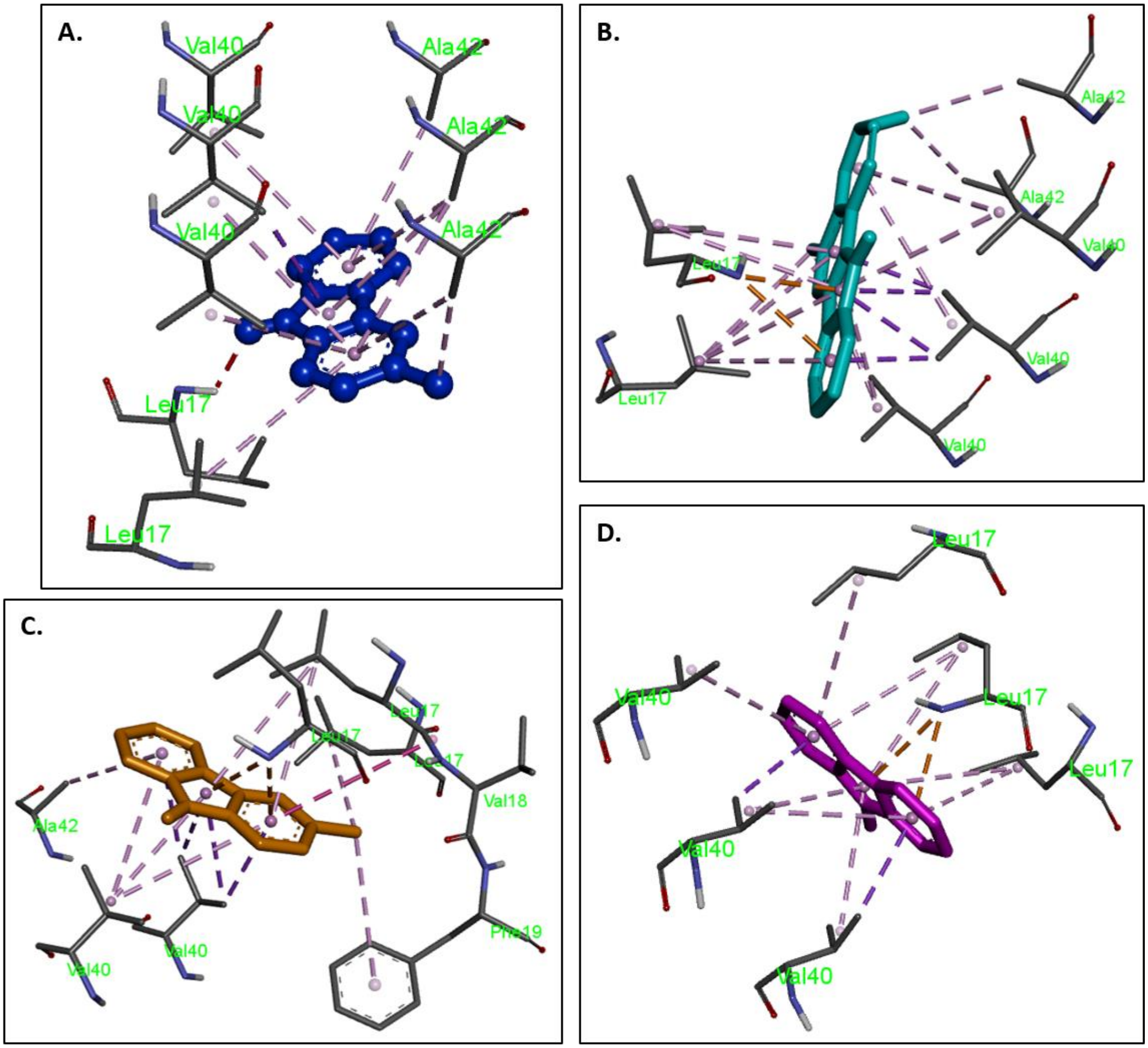

Figure S6: Binding interaction of ligands in 3D structure of the protein (8EZE). (A) murrayanol (dark blue), (B) mahanimbine (sky blue), (C) murrayafoline-A (orange), and (D) 9-methyl-9H-carbazole-2-carbaldehyde (pink).

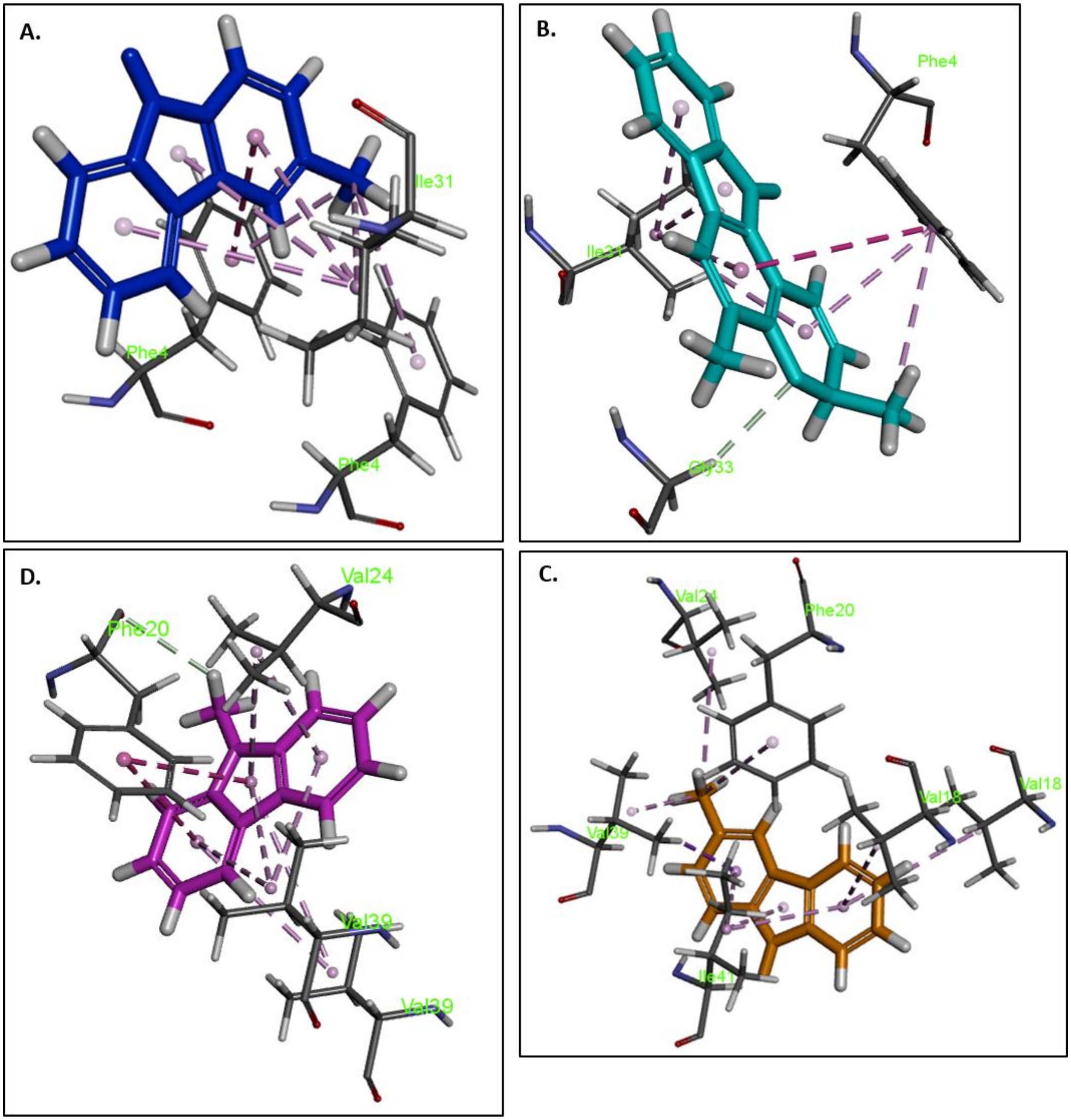

Figure S7: Binding interaction of mahanimbine and tacrine to AChE (7XN1). **A**. 2D structure of tacrine binding. **B**. 2D structure of mahanimbine binding. **C**. 3D structure of tacrine and mahanimbine binding to the same site of AChE protein.

Tacrine is a known non-competitive inhibitor for AChE binding at the PAS site of the protein. Our *in vitro* results depict non-competitive inhibition for mahanimbine, which is comparable to tacrine, since both compounds bind at the PAS site of AChE. The PAS is present at the entrance of the active site of AChE but is not a part of the catalytic triad. It has a key role in guiding ACh towards the active site and interacts with ligands that could block the substrate access or could influence enzyme conformations. The docking of mahanimbine at the PAS site could still show non-competitive inhibition, as the substrate can still bind to the active site without any hindrance.

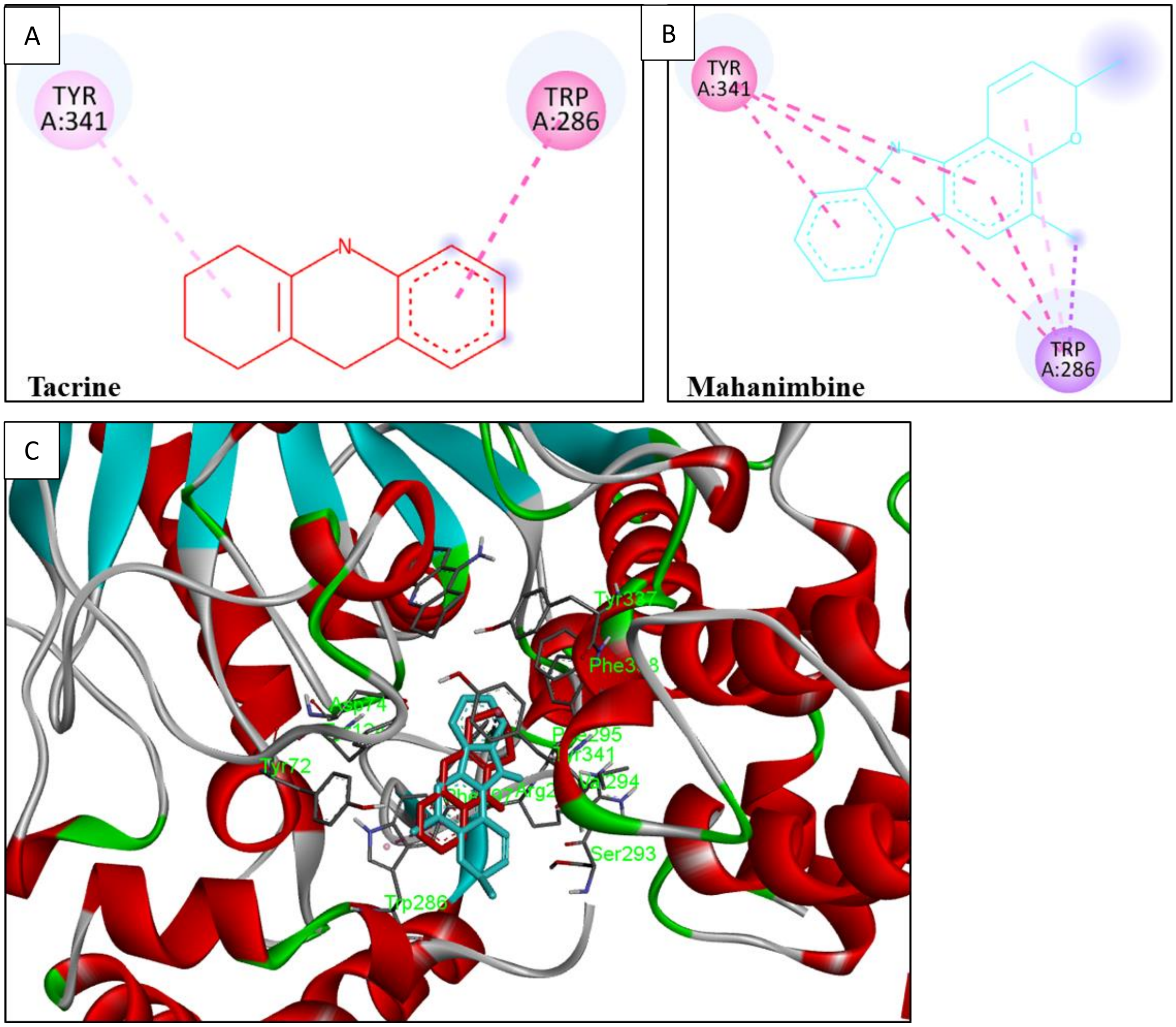

Supplement: Supplementary file 1 [file molecules-30-03138-s001.zip › molecules-3779461-supplementary.pdf]
